# Care pathways across the primary-hospital care continuum: using the multi-level framework in explaining care coordination

**DOI:** 10.1186/1472-6963-13-296

**Published:** 2013-08-06

**Authors:** Sabine Van Houdt, Jan Heyrman, Kris Vanhaecht, Walter Sermeus, Jan De Lepeleire

**Affiliations:** 1 Department of General Practice, Katholieke Universiteit Leuven, Kapucijnenvoer 33 blok J, box 7001, 3000 Leuven, Belgium; 2 Centre for Health Services and Nursing Research, Katholieke Universiteit Leuven, 3000 Leuven, Belgium; 3 Western Norway Research Network on Integrated Care, Haugesund, Norway

**Keywords:** Critical pathways (mesh), Care pathways, Multi-level framework, Coordination, Primary health care (mesh), Hospitals (mesh), Quality of care, Multiple case study

## Abstract

**Background:**

Care pathways are widely used in hospitals for a structured and detailed planning of the care process. There is a growing interest in extending care pathways into primary care to improve quality of care by increasing care coordination. Evidence is sparse about the relationship between care pathways and care coordination.

The multi-level framework explores care coordination across organizations and states that (inter)organizational mechanisms have an effect on the relationships between healthcare professionals, resulting in quality and efficiency of care.

The aim of this study was to assess the extent to which care pathways support or create elements of the multi-level framework necessary to improve care coordination across the primary - hospital care continuum.

**Methods:**

This study is an in-depth analysis of five existing local community projects located in four different regions in Flanders (Belgium) to determine whether the available empirical evidence supported or refuted the theoretical expectations from the multi-level framework. Data were gathered using mixed methods, including structured face-to-face interviews, participant observations, documentation and a focus group. Multiple cases were analyzed performing a cross case synthesis to strengthen the results.

**Results:**

The development of a care pathway across the primary-hospital care continuum, supported by a step-by-step scenario, led to the use of existing and newly constructed structures, data monitoring and the development of information tools. The construction and use of these inter-organizational mechanisms had a positive effect on exchanging information, formulating and sharing goals, defining and knowing each other’s roles, expectations and competences and building qualitative relationships.

**Conclusion:**

Care pathways across the primary-hospital care continuum enhance the components of care coordination.

## Background

Patients with chronic diseases often need long-term, complex care from different caregivers who are becoming more and more interdependent for their overall quality of care [1]. This leads to an increasing need for improved care coordination [2]. To ensure health and to enable effective care, the care should be organized around medical conditions running across the boundaries of settings, organizations and healthcare professionals [3].

In the international literature, many strategies for coordinating care are described. The choice of a coordination strategy depends upon the degree of uncertainty and the complexity of care [2,4]. These strategies do not always lead to the desired outcome [2,5-8]. More clarity about care coordination and the underlying concepts is needed to develop an effective strategy in daily practice [2].

Care pathways are often put forward as a possible strategy to improve care coordination in care situations with low uncertainty and low complexity [2]. A care pathway is defined as a complex intervention for the patient-care management of a well-defined group of patients during a well-defined period of time [9]. Care pathways can have a positive effect on outcome indicators for specific patient population [10-12]. Originally developed for high volume patients in hospitals, there is a growing interest to extend care pathways into primary care and community health [13]. Evidence is sparse about the relationship between care pathways and care coordination [14,15].

In Belgium, care pathways are developed as quality-improvement projects initiated by healthcare professionals after experiencing a gap in current practice (bottom-up) or imposed by the management (top-down) [16]. The principal outcomes of care pathways are both effectiveness and efficiency indicators. In 2000 the Belgian Dutch Clinical Pathway Network, a social capital network, was started by University of Leuven to support the development of care pathways [17]. During the past 10 years, care pathways were mainly developed in acute, mental health and rehabilitation hospitals. Recently, care pathways are also developed in primary care and between primary and hospital care. At this moment, more than 110 organizations are affiliated with more than 1300 projects [16].

The aim of this study was to assess the extent to which care pathways can support or create elements necessary to improve care coordination between primary and hospital care.

### Theoretical framework

An in-depth analysis of existing theoretical frameworks for the study of care coordination led to the identification of 14 key concepts of care coordination [18].

The most comprehensive theoretical framework exploring care coordination across organizations was the multi-level framework [19]. The multi-level framework included all identified key concepts of care coordination, except external factors, cultural factors and team outcomes. The multi-level framework is frequently applied in healthcare settings and is an extension of the relational coordination framework [20], which describes care coordination within the context of an organization.

The multi-level framework states that organizational mechanisms have an effect on the relationship between healthcare professionals, resulting in quality and efficiency of care. Key concepts identified in the multi-level framework as organizational mechanisms are “structure”, “knowledge and technology”, “administrative operational processes” and “task characteristics”. The “structure” consists of the physical and organizational aspects that support and direct the provision of care. “Knowledge and technology” are available skills, expertise, training, and information technology. “Administrative operational processes” contain standardization or adaptation during a personal interaction between healthcare professionals or during joint planning and decision making. “Task characteristics” include the degree to which team members depend on each other, the complexity and uncertainty of the task. Healthcare professionals experience a need to exchange information and to coordinate care depending on the available organizational mechanisms [19]. The perceived or evaluated “need for coordination” is the fifth identified key concept.

The identified organizational mechanisms have an effect on the relational coordination. This concept emphasizes the importance of four key concepts, including the “exchange of information”, “goals”, “roles” and “quality of relationship” [20]. The “exchange of information” addresses the transfer of information, ideas and opinions in a frequently, accurately, timely and problem-solving manner. “Goals” consider the importance of setting common goals, sharing these goals and assuring collective ownership of these goals. “Roles” focus on the definition of roles and the awareness of each other’s roles. “Quality of relationship” promotes mutual respect and high quality collaboration.

**Figure 1:**
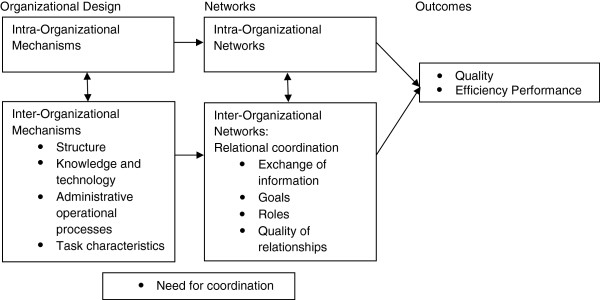
The multi-level framework.

By using the same organizational mechanisms both within and between organizations, networks are even more strengthened, resulting in more quality and efficiency of care [19]. Quality and efficiency are considered as important “patient outcomes” and “inter-organizational outcomes”. The multi-level framework is illustrated in Figure 1.

## Methods

A multiple case study design [21] was used to provide an in-depth analysis of the relationship between care pathways and care coordination. Data were gathered using mixed methods, including structured face-to-face interviews, participant observations, documentation and a focus group. Multiple cases were analyzed performing a cross case synthesis to strengthen the results by confirming the findings in other cases or by understanding how, why and in which circumstances care pathways improve care coordination or not. This study does not provide data about the effectiveness of care coordination (box 3 “outcomes” of Figure 1).

### Cases

Five local community projects were selected. These local community projects experienced a problem, gap or need across the primary-hospital care continuum for patients with breast or prostate cancer. Both local community projects developing a new care pathway and local community projects evaluating an existing care pathway were selected. The local community projects were located in different regions in Flanders in order to analyze the effects of the local organization of care, the local habits and the local regulations.

Of the five selected local community projects, two evaluated an existing care pathway for patients with prostate cancer. The first local community project was experiencing a need to evaluate the existing care pathway because of a changed surgical procedure which had different implications for primary care. The second local community project had developed a care pathway between primary and hospital care in 2005. After implementation of the care pathway, they were still experiencing a lack of transparency in the primary care processes. Both local community projects had experienced difficulties with an initial lack of representation of all healthcare professionals involved and with the implementation of the care pathway in primary care.

Three local community projects developed a care pathway for patients with breast cancer. One local community project started in 2002 with representatives from hospital specialists and general practitioners to redesign the follow-up of patients. Due to the difficulties they encountered, such as barriers to implementing the agreements made in daily practice, they changed their focus to the surgical phase. By limiting the targeted patient group in time from 5 years to several months, they expected that it would be easier to make agreements and test them in daily practice. Two local community projects developed a new care pathway for patients with breast cancer. These projects were carried out by two different hospitals involving primary care. As the two hospitals were located nearby, some primary healthcare professionals and organizations worked with both hospitals. In each of these local community projects, a workgroup with representatives from the hospital, the primary care and the patient associations was created. A steering group including all the primary care representatives of these two local community projects was established to observe the development of one care pathway for primary care. Table 1 presents some key features of the selected cases.

**Table 1:** Overview of selected community projects

**Case**	**Region**	**Focus**	**Patients / year**	**Start**	**Composition multidisciplinary group**	**Core staff**
1	1	Patients treated with a prostatectomy from first appointment with specialist till post-surgical control	200	2005	Representatives of hospital (n = 6) and all primary care services involved (n = 18)	Staff members of hospital, home care service and SEL^a^, all members of core staff changed during project
2	2	Patients referred to specialist for prostatectomy till follow-up	250	2005	Representatives of hospital (n = 1) and primary care (n = 5)	Staff of a home care service
3	2	Initial period “follow-up for patients with breast cancer” changed into “from referral till second post-op consultation”	200	2002	Specialists (n = 3), general practitioners (n = 3) and a specialized nurse since September 2008	1. Staff member of hospital and researcher; 2. General practitioner and specialist
4	3^b^	Surgical breast care patient from discharge from hospital till start of after treatment	160	2006	Representatives of hospital (n = 9) and primary care, including patient representatives (n = 16)	1. Staff member of hospital and SEL^a^; 2. Specialist, general practitioner and staff member of hospital
5	4^b^	Surgical breast care patient from discharge from hospital till start of after treatment	200	2006	Representatives of hospital (n = 10) and primary care including patient representatives (n = 16)	Staff member of hospital and SEL^a^

^a^SEL provides a platform of consultation to assist and extend home care, beyond the boundaries of the own organization, office or discipline.

^b^Care pathways in these cases were developed in cooperation with different hospitals but with partially overlapping primary care.

In the literature, many methodologies are described for developing, implementing and evaluating care pathways [2,9,22]. In Belgium, a 30-step scenario plan has been elaborated for developing, implementing and evaluating care pathways [23]. This step-by-step plan incorporates the basic principles found in all appropriate methodologies [9]. The selected local community projects started with this existing step-by-step plan [23], but were free to apply the methodology to their own needs and expertise.

### Data collection

The five local community projects were followed from September 2006 till mid-2010. A mixed method research design was used, including structured face-to-face interviews, participant observations, documentation and a focus group.

1. **Structured face-to-face interviews** were performed at the start of this study with representatives of all the local community projects [24]. Missing information was gathered by additional telephone interviews. The interviews were transcribed verbatim. The purpose of these interviews was to obtain a detailed understanding of the current situations and of the local opportunities, constraints and expectations.

2. A researcher followed the local community projects during the whole period of data collection through **(participant) observation**[24,25]. Collected data included date, event, initiator, other participants, goal, other issues raised, agreements made, remaining questions, strategy to answer these questions and references to relevant documents. Both the researcher and the representatives of each of the local community projects made notes of every activity.

3. **Documentation** related to the development process was also gathered during the whole period of data collection: email communication, the agendas and minutes of meetings, and patient leaflets. These data were mainly used to clarify, validate or refute data from the (participant) observation.

4. Finally, each local community project was described in a local community project report, using data from the interviews, (participant) observation and documentation. These reports consisted of a historical analysis involving the objectives, the progress made and the important lessons learned. These reports were read and, if necessary, completed and corrected by representatives of these local community projects. Based on these written reports, a number of themes with accompanying statements were formulated by the research team. These themes were used to structure a **focus group** with representatives from the local community projects and patients’ associations who came together to reflect on their experiences. This focus group was led by one of the researchers. The topics discussed were: 1) the methodology of care pathways, 2) factors influencing or influenced by the development and implementation of the pathways. A second researcher recorded the verbal and nonverbal communication. The focus group was digitally recorded and transcribed verbatim.

### Analysis

The five local community projects served as data sources for a cross-case synthesis to determine whether the available empirical evidence supported or refuted the theoretical expectations and evidence from the literature. The cases were compared and analyzed to search for patterns, similarities and differences across the cases [21]. The data were thematically analyzed by one researcher (VHS). Nvivo 8 was used to assist with coding, sorting and retrieval. The results were reflected upon with the other members of the research team (DLJ, HJ, SW). Confirmation of the results was also gained by member checking. Representatives from the local community projects (both from primary and hospital care) and patients’ associations regularly met to exchange and reflect upon the results on the basis of their experiences.

### Ethics statement

This study was approved by the Ethics Committee of the University Hospital in Leuven (B32220096079, S51552) and site specific approval from the Jessa Hospital in Hasselt (09.02/onchae09.02), the Hospital East Limburg in Genk (07/077) and the University Hospital in Leuven (B32220108500, S52182). All participants were informed about the study. All patients included in this study signed an informed consent form.

## Results

**Figure 2:**
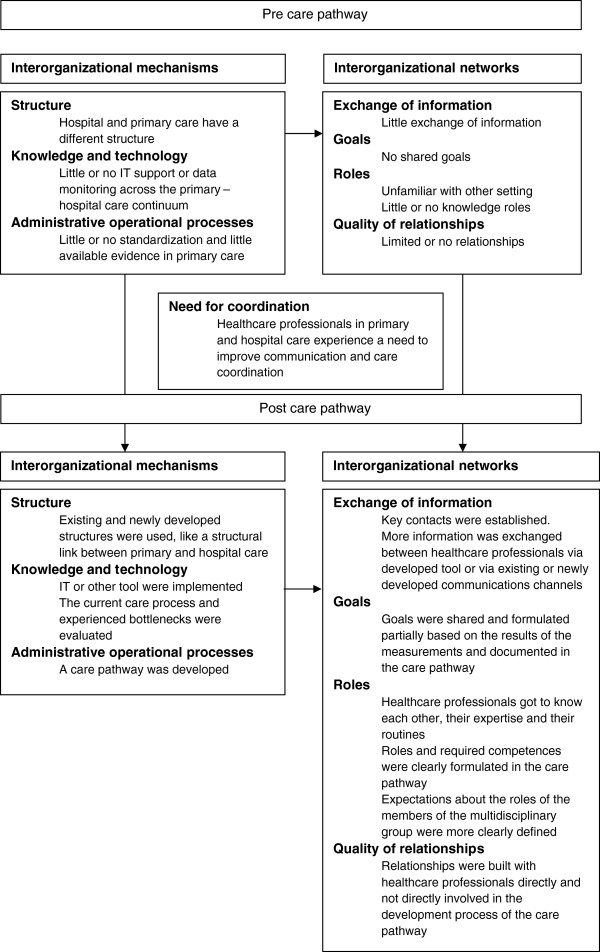
Relationship between care pathways across the primary-hospital care continuum and care coordination.

The results focus on the existing elements of the multi-level framework and the impact of a care pathway on these elements. The results are illustrated with anonymous fragments of the collected data. These fragments were originally recorded in Dutch. The results are presented in Figure 2.

### Inter-organizational Mechanisms

Inter-organizational mechanisms of the multi-level framework, identified in the local community projects as influencing the networks between healthcare professionals were the “structure”, “knowledge and technology” and “administrative operational processes”. No data was found on “task characteristics”. Local community projects evaluating an existing care pathway already had taken initiatives to enhance inter-organizational mechanisms and networks during the development process of the first version of the care pathway. These initiatives are elaborated on in care pathways. No differences were found at this point between the two diagnostic groups or between regions.

#### Structure

Primary and hospital care were two different entities. Primary care consisted of a number of geographically spread, small practices, services and self-employed healthcare professionals without a hierarchical structure. Because of the existing freedom of choice of patients concerning their primary healthcare professionals, primary healthcare teams varied per patient. As a result primary healthcare professionals worked together with large numbers of other primary healthcare professionals.

*I would like to refer to the freedom of choice of the patient, which is legally defined in the Act of August 22, 2002 concerning the rights of patients. […] There is often already a network of primary healthcare professionals who surround the patient. […] I don’t think it’s a good idea then to replace people, certainly if there is already a relationship of trust. (Focus group, 1–5, 2, representative of a patients’ association, 142)*^*a*^.

Healthcare professionals in hospital care and even some primary healthcare professionals were unfamiliar with the structure of primary care. Hospital care was geographically located to one place, consisted of fixed teams and had more hierarchical structure. Nevertheless, this structure was in fact just as vague in the eyes of the primary healthcare professionals.

They say that primary care is fragmented, but hospital care is just as fragmented in the eyes of the primary care providers. (Focus group, 2, 1, primary care, nurse, 133).

#### Knowledge and technology

There was little or no IT support or data-monitoring (e.g. performance measuring) across the primary and hospital care - continuum. Primary care also had less validated instruments and opportunities for measuring the team, organization and experiences of patient and family.

An instrument was developed based on patients’ experiences and relevant parts of existing questionnaires. Face and content validity was achieved.

*During their hospital stay, the study was explained to the patients who met the inclusion criteria. If they agreed to participate, they were asked to sign an informed consent agreement. (Documentation, 2, research protocol, 20100322)*^*b*^*.*

#### Administrative operational processes

There was little or no standardization of the care process. A certain degree of standardization was required before primary and hospital care can be linked up.

I have the feeling that we’ve done this process three times. We’ve learnt that we first have to standardize our own process. Next, the process in primary care needs to be more transparent. And then finally, there is the connection with the hospitals. You have to do this process in three steps. (Focus group, 2, 1, primary care, nurse, 27).

In comparison with hospital care, little evidence existed relating to the care processes in primary care.

In primary care there is less evidence available, because less research was performed in the past. (Focus group, 2, 1, primary care, nurse, 136).

### Inter-organizational networks

The lack of structure, knowledge and technology, and administrative processes between primary and hospital care was also found in the limited relationships that existed between primary care providers and hospital care providers for the exchange of information. Healthcare professionals in primary and hospital care do not know each other, or their roles, expertise, procedures and organizations.

*During an information meeting in the two local community projects starting to develop a care pathway, each setting presented itself. The aim of this presentation was to get to know each other. The presentation included how each setting is organized, as well as important procedures, regulations and habits. The hospitals also presented their developed (intramural) care pathway. (Participant observation, 4, 20061123 and 5, 20061127)*^*c*^.

### Need for coordination

Healthcare professionals experienced a need to improve communication and care coordination by developing a care pathway or evaluating an existing care pathway.

*An added value of developing care pathways is the evaluation of information flows. A care pathway always leads to who gets what information when. (Focus group, 1, 4, hospital, quality coordinator, 103)*.

### Care pathways

In each local community project a care pathway across the primary – hospital care continuum was developed or evaluated for patients with breast or prostate cancer. The in-depth analysis of these local community projects learned that care pathways between primary and hospital care enhance inter-organizational mechanisms and network components of the multi-level framework. Local community projects evaluating an existing care pathway already had taken initiatives during the development of the first version of the care pathway. We also found regional differences. No differences were found between the two diagnostic groups.

#### Structure

The process of developing a care pathway led to the use of newly constructed and existing structures and communication channels. The construction of a good structure, taking into account existing structures and communications channels was experienced as very important in the local community projects. Local community projects that did not invest enough time and energy in the beginning came to experience they needed to do this later in the project.

*After the information meeting organized in the local community projects that were starting to develop a care pathway, initiatives were taken during the following five months to create a structure and communication network with representatives from all the disciplines involved, including patients’ associations. (Participant observation, 4–5, 200611–200704)*.

*We’ve invested a lot of energy in the construction of the structure in order to find the right people to participate. […] We have two motivated GP’s who give feedback to their peers. We notice that it comes to life and we get positive responses. It takes time, but it is necessary. If you go too fast in the first part, you’ll mortgage a part of your process. (Focus group, 4–5, 8, primary care, SEL*^*d*^*coordinator, 5).*

In all local community projects a structural link was established to build a bridge between primary and hospital care. This link was already established in local community projects evaluating an existing care pathway.

*A Clinical Pathway Steering Committee was established with the following objectives: 1) to follow the methodology, development, implementation and monitoring of care pathways, and 2) to provide a contact for hospital care. (Documentation, 1, 3, 200605)*.

This structural link needed to be closely related to the daily practice of healthcare professionals, including sufficient expertise of the setting (e.g. primary or hospital care), local procedures and sensitivities. All healthcare professionals and organizations involved needed to perceive this structural link with an acceptable degree of neutrality. ‘Acceptable degree of neutrality’ was understood to imply that all involved have (sufficient) confidence that this structural link pursues the common good. Some persons or organizations have this neutrality de facto, others can obtain it. Obtaining neutrality requires the willingness of others and the passage of time. The role of this structural link is to assist and guide local healthcare professionals during the development, implementation and evaluation of the care pathway, both in terms of having the necessary expertise in the methodology of care pathways and in terms of providing administrative and logistic support.

*To be able to meet the expectations, primary healthcare professionals, such as general practitioners, physiotherapists and independent nurses, need support […] Support includes administrative relief, follow-up, making notes, informing people, …¨ […] I think this is linked with the fact that a neutral organization can more easily bring all the partners involved together, while another organization may have a number of barriers to overcome. (Focus group, 1–5, 2, representative of a patients’ association, 19)*.

*It isn’t easy for the SEL. There is insufficient staff and they have a lack of the necessary expertise. I’m not medically educated and I don’t know much about care pathways. Although I now know more than when I started working for the SEL. (Focus group, 1, 5, primary care, SEL coordinator, 24)*.

An example of the use of existing structures was the explicit question to the representatives of primary care in two local community projects how soon they could inform or ask feedback from their peers. Members of primary care checked which structures and information channels existed to fulfill these roles and expectations. Moreover, by asking this question explicitly, the roles of and expectations towards members of the multidisciplinary group were clearly communicated. It became clear that each discipline could disseminate information or gather feedback within a month.

*It was important for us to have a mandate through the organizations. […] We actively asked in which period each discipline could inform their colleagues. People really started thinking about how to inform their colleagues: via an email system, monthly meetings […]. By asking this, you are in fact pointing out to the representatives their responsibilities. (Focus group, 4–5, 8, primary care, SEL coordinator, 41)*.

The use of existing and newly constructed structures had a positive influence on the networks between primary and hospital care: healthcare professionals got to know each other and their routines; structures and partnerships were developed; roles were clearly defined; and key contacts were made.

*During the development of the care pathway, we made contact with primary care. We learnt that it was not the pathway developed that was the most important thing, but rather the fact that you have a contact person and that good agreements are made. (Focus group, 1, 4, hospital, quality coordinator, 33)*.

Moreover, these developed networks were not restricted to the healthcare professionals directly involved in the development process of the care pathway. Two local community projects succeeded in using the existing and newly developed structures to create a sense of “ownership” with all healthcare professionals. The sense of “ownership” was promoted by regularly informing all healthcare professionals, by increasing the feeling that all healthcare professionals were represented and by measuring and reporting about the results. This sense of “ownership” among all healthcare professionals was considered important in motivating healthcare professionals to comply with the agreements made. Although feedback and information were necessary, it was important not to burden the colleagues.

Although it is the entire group of people who work together to develop a care pathway, our experience is that it is always the same people who come together and give feedback. Others don’t feel a sense of ownership of the care pathway. (Focus group, 1, 4, hospital, quality coordinator, 14).

*I’m thinking, “what would be an added value for me, if it were developed externally”? If I felt involved in some way, if I knew that a nurse was involved who thinks critically about it, so it would be linked to daily practice. (Focus group, 2, 1, primary care, nurse, 181)*.

Regional regulation and habits in these two local community projects supported the use of (new) structures, networks and the creation of a sense of “ownership”. Primary care in these local community projects developed about 15 years ago a “code”, signed by all primary healthcare professionals in the region. This code included principles for multidisciplinary team working like comprehensive care involving the patient and all necessary primary and hospital care professionals; identifying and communicating problems; and making agreements between healthcare professionals. These principles were translated throughout the years in regional habits. This regional “culture” enhanced inter-organizational mechanisms and networks.

#### Knowledge and technology

IT was considered to be an important tool for implementing and evaluating care pathways in daily practice. According to the representatives of all local community projects, the future of care pathways is determined by the potential support of and by their integration into existing electronic systems.

*The future of care pathways depends upon IT. That’s the message I’ve been receiving from my colleagues. If it isn’t supported electronically, then it doesn’t work. (Focus group, 3, 7, primary care, general practitioner, 225)*.

More specifically, these representatives had in mind the provision of information through a central website, the integration of the agreements made into their own electronic files, and the electronic exchange of information between the healthcare professionals involved.

*For me, a care pathway is a tool that I can use only if it is integrated into the software of my electronic files. (Focus group, 3, 7, primary care, general practitioner, 184)*.

Since electronic information exchange was not currently possible, the local community projects developed a booklet or leaflet as a temporary solution. These tools were already developed in local community project evaluating an existing care pathway. The aim of these documents was to follow the patients throughout the care process, to provide information to the patients and healthcare professionals involved, to create the possibility to exchange information and to empower the patient. The documents also contained information about the expected roles of healthcare professionals involved (based on current guidelines, experiences or agreements made between healthcare professionals).

The brochure is an aid for all who are involved: the many health care professionals, both in hospital care and in primary care, as well as the patients. If you don’t have a system for communicating with these three, it is very difficult. (Focus group, 3, 7, primary care, general practitioner, 100).

Nevertheless, all local community projects experienced problems with the implementation of this leaflet or booklet. Possible influential factors included: structural aspects (the format, the moment that the document was given, the double registration of data), lack of information about the objectives and the importance of the document, lack of ownership by the patient and / or the health care professionals involved and the ability or the motivation of the patient to realize the expected role. Some local community projects experimented with providing certain required information via standardized documents, including referral letter, discharge letter and prescription to exchange information and expectation about the role of healthcare professionals involved.

In all local community projects data were gathered during the development process of the care pathway about the current care process and the experienced bottlenecks. The evaluation of the care process led to objectifying certain assumptions, discovering blind spots, motivating more reserved healthcare professionals and creating greater acceptance.

*The results of the measurements were very important because they increased the awareness of the doctors and staff members in the hospitals that the patients experience problems when they go home. The doctors and staff are not always aware of these problems, and the patients don’t mention them during consultation. You get more response if you can illustrate these problems with the results of interviews with patients. (Focus group, 1, 4, hospital, quality coordinator, 71)*.

*If you start from certain assumptions, the danger exists that you will focus on the biggest bottlenecks in the process as you experience it. If you measure, then you come to know the most important bottlenecks, or else you get a confirmation of what you already knew. Measurements provide an added value for your activities: you know you are on the right track and nuances can be made. (Focus group, 5, 3, hospital, care manager, 67)*.

Two of the three local community projects evaluating an existing care pathway gathered data during the development of the first version of the care pathway. The third local community project also experienced a need to gather data, but lacked support and expertise.

#### Administrative operational processes

Standardization of the care process by developing a care pathway across the primary and hospital care continuum led to the definition of goals, roles and required expertise. These were formulated as concretely as possible. All local community projects evaluating a care pathway had struggled with formulating clear goals, roles or competences. They experienced this vagueness as a bottleneck and later clarified or adjusted these.

Due to the existing freedom of choice, healthcare professionals in all local community projects experienced difficulties in referring patients to primary healthcare professionals with specific competences. Four strategies were distinguished for ensuring that, when necessary, patients consulted healthcare professionals with the required competences. The required competences needed to be clearly defined in the care pathway. Moreover, sufficient and explicit information needed to be provided to the patients and the healthcare professionals involved, including the general practitioner. The role and expectations of each healthcare professional had to be clearly defined: each healthcare professional was also responsible for referring patients when necessary. A social map with all the healthcare professionals and their competences needed to be developed.

*Representatives of the hospitals noted that they can’t refer patients only to a limited group of healthcare professionals because they feel like excluding other healthcare professionals. Moreover, they emphasize that some actions, like shoulder exercises after mastectomy, can also be performed by non-specialized healthcare professionals. (Documentation, 1–5, 1, 20070913)*^*d*^.

*I do think that certain competences are necessary. During the consultation, a general practitioner can give his patient a list of regional healthcare professionals and their competences. (Focus group, 1–5, 2, representative of a patients’ association, 142)*.

## Discussion

The development of a care pathway across the primary – hospital care continuum, supported by a step-by-step scenario, led to the enhancement of inter-organizational mechanisms and network components of the multi-level framework. Local community projects evaluating an existing care pathway already had taken initiatives to enhance these components during the development of the first version of the care pathway. Regional differences had an influence on the use of (new) structures, networks, the creation of a sense of “ownership” and data gathering. No differences were found between the two diagnostic groups.

The process of developing a care pathway led to the use of existing and newly developed structures. The construction of a good structure, taking into account existing structures and communication channels was considered even more important than the development of the care pathway per se: key contacts were made, healthcare professionals got to know each other and partnerships were built. Currently hospital care in Belgium is more structured than primary care. Nevertheless it is widely accepted that a strong primary care system can improve the coordination and responsiveness in health care [26]. Healthcare systems recognizing the importance of primary care to coordinate care, who are regionally organized (including hospital care) and have their own resources to respond to their experienced need, will have more structural support available for developing and implementing care pathways between primary and hospital care. Without these necessary preconditions, it will be up to “early innovators” to change things in the existing systems and frameworks.

General practitioners are often considered to be the structural link for coordinating between primary and hospital care for the individual patient. However, the electronic health records that are needed for the exchange of relevant information and for providing the necessary links are often lacking [27].

The support of and integration into IT applications was considered to be another essential element for the future of care pathways. A systematic review of the impact of eHealth on the quality and safety of healthcare concluded that there is a large gap between the postulated and the empirically demonstrated benefits of eHealth technologies [28]. Another systematic review, studying the effects of health information technology, found in 62% of the studies that one or more aspects of the care improved with no aspects being aggravated, 30% of the studies had mixed results, and 8% did not produce any positive results. An analysis of these negative studies teaches us that the human factor is very important, more specifically the satisfaction people gain by using these electronic systems [29].

All the local community projects have experienced problems with the implementation of the developed booklet or leaflet in daily practice. In a case study about the development of a patient safety care pathway, separate tools were developed to represent the care pathway, to coordinate healthcare professionals’ activities and to account for action to meet the multiple purposes and the multiple stakeholders of the care pathway [30]. Developing different documents could resolve the current difficulties being experienced with implementation.

The local community projects started with the existing 30-step care pathway methodology [23] to develop, implement and evaluate a care pathway across the primary –hospital care continuum. The importance of including all relevant stakeholders and the benefit of continuous and reflective learning was also demonstrated in a study about the development of a care pathway across different settings and independent disciplines [31]. Reviewing both the evidence of and the feedback on the actual organization of the care process are two important aspects in the methodology of care pathways [9,15]. Nevertheless, little evidence exists for the purpose of formulating key interventions and outcome indicators across the primary – hospital care continuum [32-36]. A certain amount of variation is accepted, so that pathways can be tailored for particular purposes and creative solutions can be developed for managing the interdependencies of their components in particular circumstances [30]. Some variations were found related to the specific characteristics of the primary – hospital care continuum.

The experiences of these local community projects have contributed to the revision of the existing step-by-step plan into a model of seven phases [37]. Moreover, these results were translated into a blueprint for projects aimed at developing a care pathway either in primary care alone, or in primary care together with hospital care.

The inter-organizational mechanisms had an effect on the relationships between the healthcare professionals involved. Timely and accurate communication and information exchange between the primary and the secondary healthcare professionals involved is often inadequate or even lacking [38]. Poor communication between agencies and a lack of understanding of each other’s roles and responsibilities are barriers to coordinating multi-agency practice [39]. Staiger [40] formulates the future challenges in terms of redefining the roles and supporting the staff in their efforts to develop effective strategies to enhance communication, collaboration and coordination between the acute and the community health sectors. The local community projects used a variety of strategies to involve all healthcare professionals. Grol [41] emphasizes the importance of using a multifaceted approach via existing channels and structures to implement Evidence-Based Guidelines for clinical practice.

The five selected local community projects developed, implemented and evaluated a care pathway for patients with surgery after a diagnosis of breast or prostate cancer. The conclusions formulated were not tested for other pathologies. Since our focus was on the process of developing, implementing and evaluating care pathways, the conclusions formulated are likely applicable to other pathologies.

The care pathways developed in the five local community projects were guided by a 30-step scenario and had partial support (academic and financial). These additional supports have a beneficial influence on the findings of this study.

Many strategies were used to ensure validity and reliability [21]. Multiple data sources were used in the five local community projects, so that the results could be confirmed both within and between cases. The multi-level framework guided the analysis. The results were substantiated with empirical evidence and discussed with other researchers and members of the local community projects.

The development of a care pathway across the primary-hospital care continuum, supported by a step-by-step scenario, led to the enhancement of inter-organizational mechanisms and network components of the multi-level framework. To support the development of care pathways, policy makers need to provide the necessary structure so qualitative relationships can be built. The integration of care pathways into current IT applications is essential, taking into account the needs and expectations of healthcare professionals. More research is required to study the effects of care pathways across organizational boundaries on the coordination and quality of care.

## Conclusion

Care pathways across the primary-hospital care continuum enhance the components of care coordination. The development of the care pathways, supported by a step-by-step scenario, strengthened inter-organizational mechanisms and network components of the multi-level framework.

## Endnotes

^a^ (data source, nr project, nr participant, working area of participant (not applicable for representatives of patients’ associations), background, nr quotation)

^b^ (data source, nr project, type doc, date)

^c^ (data source, nr project, date)

^d^ SEL provides a platform of consultation to assist and extend home care, beyond the boundaries of the own organization, office or discipline.

## Competing interests

There was no financial or non-financial conflict of interest.

## Authors’ contributions

SVH, JH, WS and KV contributed to the conception and design of this study. SVH carried out the structured face-to-face interviews, the (participant) observation, the collection of relevant documentation and participated as an observer in the focus group. SVH also analysed and interpreted all data and drafted the manuscript. JH participated as an observer in the focus group and helped with the interpretation of all the data. WS moderated the focus group and helped with the interpretation of all the data. JDL helped with the interpretation of all the data. All authors have critically revised the manuscript and approved the final version.

## Pre-publication history

The pre-publication history for this paper can be accessed here:

http://www.biomedcentral.com/1472-6963/13/296/prepub

## References

[B1] Nolte E McKee M Nolte E, McKee M Caring for people with chronic conditions: an introduction Caring for people with chronic conditions. A health system perspective 2008 Berkshire: Open University Press

[B2] McDonald KM Sundaram V Bravata DM Lewis R Lin N Kraft S McKinnon M Paguntalan H Owens DK Shojania KG, McDonald KM, Wachter RM, Owens DK Care coordination Closing the quality gap: a critical analysis of quality improvement strategies 2007 Rockville, MD: Agency for Healthcare Research and Quality 20734531

[B3] Porter ME Teisberg EO How physicians can change the future of health care JAMA 2007 297 10 1103 1111 10.1001/jama.297.10.1103 17356031

[B4] Alter C Hage J Organizations working together 1993 Newbury Park, California: Sage Publications

[B5] Barbieri A Vanhaecht K Van HP Sermeus W Faggiano F Marchisio S Panella M Effects of clinical pathways in the joint replacement: a meta-analysis BMC Med 2009 7 32 10.1186/1741-7015-7-32 19570193 PMC2715423

[B6] Hughes DC Luft HS Managed care and children: an overview Future Child 1998 8 2 25 38 10.2307/1602672 9782648

[B7] Smith S Allwright S O’Dowd T Effectiveness of shared care across the interface between primary and specialty care in chronic disease management Cochrane Database Syst Rev 2007 CD004910 10.1002/14651858.CD004910.pub2 17636778

[B8] Valesco-Garrido M Busse R Hisashige A Are disease management programmes (DMPs) effective in improving quality of care for people with chronic conditions? 2003 Copenhage: WHO Regional Office for Europe

[B9] Vanhaecht K Panella M van Zelm R Sermeus W An overview of the history and concept of care pathways as complex interventions International Journal of Care Pathways 2010 13 117 123

[B10] Hensen P Ma HL Luger TA Roeder N Steinhoff M Pathway management in ambulatory wound care: defining local standards for quality improvement and interprofessional care Int Wound J 2005 2 2 104 111 10.1111/j.1742-4801.2005.00098.x 16722861 PMC7951494

[B11] Rotter T Kugler J Koch R Gothe H Twork S van Oostrum JM Steyerberg EW A systematic review and meta-analysis of the effects of clinical pathways on length of stay, hospital costs and patient outcomes BMC Health Serv Res 2008 8 265 10.1186/1472-6963-8-265 19094244 PMC2632661

[B12] Rotter T Kinsman L James E Machotta A Gothe H Willis J Snow P Kugler J Clinical pathways: effects on professional practice, patient outcomes, length of stay and hospital costs Cochrane Database Syst Rev 2010 3 CD006632 10.1002/14651858.CD006632.pub2 20238347

[B13] Campbell H Hotchkiss R Bradshaw N Porteous M Integrated care pathways BMJ 1998 316 7125 133 137 10.1136/bmj.316.7125.133 9462322 PMC2665398

[B14] Atwal A Caldwell K Do multidisciplinary integrated care pathways improve interprofessional collaboration? Scand J Caring Sci 2002 16 4 360 367 10.1046/j.1471-6712.2002.00101.x 12445105

[B15] Brown JB Sutton L A neurological care pathway for meeting the palliative care needs of people with life-limiting neurological conditions Int J Palliat Nurs 2009 15 3 120 127 19537532 10.12968/ijpn.2009.15.3.41090

[B16] Van Gerven E Vanhaecht K Deneckere S Vleugels A Sermeus W Management challenges in care pathways: conclusions of a qualitative study within 57 health-care organizations International Journal of Care Pathways 2010 14 4 142 149 10.1258/jicp.2010.010029

[B17] Sermeus W Vanhaecht K Vleugels A The Belgian-Dutch clinical pathway network Journal of Integrated Care Pathways 2001 5 10 14

[B18] Van Houdt S Heyrman J Vanhaecht K Sermeus W De Lepeleire J An in-depth analysis of theoretical frameworks for the study of care coordination International Journal of Integrated Care 2013 13 [http://www.ijic.org/index.php/ijic/article/view/URN:NBN:NL:UI:10-1-114598] 10.5334/ijic.1068 PMC3718267 23882171

[B19] Gittell JH Weiss SJ Coordination networks within and across organisations: a multilevel framework J Management Studies 2004 41 1 127 153 10.1111/j.1467-6486.2004.00424.x

[B20] Gittell JH Coordinating mechanisms in care provider groups: relational coordination as a mediator and input uncertainty as a moderator for performance effects Manag Sci 2002 48 1408 1426 10.1287/mnsc.48.11.1408.268

[B21] Yin RK Case study research 2009 California: Sage Publications

[B22] Pearson SD Goulart-Fisher D Lee TH Critical pathways as a strategy for improving care: problems and potential Ann Intern Med 1995 123 12 941 948 10.7326/0003-4819-123-12-199512150-00008 7486490

[B23] Vanhaecht K Sermeus W Vleugels A Peeters G Ontwikkeling en gebruik van klinische paden (“clinical pathways”) in de gezondheidszorg Tijdschrift voor geneeskunde 2002 58 23 1542 1551 10.2143/TVG.58.23.5001477

[B24] Bryman A Social research methods 2004 Oxford: University Press

[B25] Mays N Pope C Qualitative research: observational methods in health care settings BMJ 1995 311 6998 182 184 10.1136/bmj.311.6998.182 7613435 PMC2550229

[B26] Gress S Baan CA Calnan M Dedeu T Groenewegen P Howson H Maroy L Nolte E Reaelli M Saarelma O Schmacke N Schumacher K van Lente EJ Vrijhoef B Co-ordination and management of chronic conditions in Europe: the role of primary care–position paper of the European forum for primary care Qual Prim Care 2009 17 1 75 86 19281678

[B27] Bodenheimer T Coordinating care–a perilous journey through the health care system N Engl J Med 2008 358 10 1064 1071 10.1056/NEJMhpr0706165 18322289

[B28] Black AD Car J Pagliari C Anandan C Cresswell K Bokun T McKinstry B Procter R Majeed A Sheikh A The impact of eHealth on the quality and safety of health care: a systematic overview PLoS Med 2011 8 1 e1000387 10.1371/journal.pmed.1000387 21267058 PMC3022523

[B29] Buntin MB Burke MF Hoaglin MC Blumenthal D The benefits of health information technology: a review of the recent literature shows predominantly positive results Health Aff (Millwood) 2011 30 3 464 471 10.1377/hlthaff.2011.0178 21383365

[B30] Allen D From boundary concept to boundary object: the practice and politics of care pathway development Soc Sci Med 2009 69 3 354 361 10.1016/j.socscimed.2009.05.002 19481321

[B31] Campbell S MacDonald M Carr B Anderson D MacKinley R Cairns S Bridging the gap between primary and secondary care: use of a clinical pathway for the investigation and management of deep vein thrombosis J Health Serv Res Policy 2008 13 Suppl 1 15 19 10.1258/jhsrp.2007.007015 18325163

[B32] Cardoso F Stordeur S Vlayen J Bourgain C Carly B Christiaens MR Cocquyt V Lifrange E Neven P Scalliet P Schobbens C Van Goethem M Villeirs G Wetenschappelijke ondersteuning van het College voor Oncologie: een update van de nationale richtlijn voor borstkanker 2010 Brussels: Federaal Kenniscentrum voor de Gezondheidszorg

[B33] Christiaens MR Vlayen J Gailly J Neven P Carly B Schobbens C Drijkoningen R Lifrange E Cocquyt V Bourgain C Villeirs G Larsimont D D’Haese S De Grève J Wetenschappelijke ondersteuning van het College voor Oncologie: een nationale richtlijn voor de aanpak van borstkanker 2007 Brussels: Federaal Kenniscentrum voor de Gezondheidszorg

[B34] Scottish Intercollegiate Guidelines Network Management of breast cancer in women: a national clinical guideline 2005 Edinburgh: NHSScotland 10.1016/j.clon.2007.06.006 17643275

[B35] Hunter KF Moore KN Cody DJ Glazener CM Conservative management for postprostatectomy urinary incontinence Cochrane Database Syst Rev 2004 2 CD001843 10.1002/14651858.CD001843.pub2 15106164

[B36] Scottish Intercollegiate Guidelines Network Management of urinary incontinence in primary care: A national clinical guideline 2004 Edinburgh: NHSScotland

[B37] Vanhaecht K Van Gerven E Deneckere S Lodewijckx C Janssen I van Zelm R Boto P Mendes R Panella M Biringer E Sermeus W The 7-phase method to design, implement and evaluate care pathways The International Journal of Person Centered Medicine 2012 2 3 341 351

[B38] Kripalani S LeFevre F Phillips CO Williams MV Basaviah P Baker DW Deficits in communication and information transfer between hospital-based and primary care physicians: implications for patient safety and continuity of care JAMA 2007 297 8 831 841 10.1001/jama.297.8.831 17327525

[B39] Tearl DK Cox TJ Hertzog JH Hospital discharge of respiratory-technology-dependent children: role of a dedicated respiratory care discharge coordinator Respir Care 2006 51 7 744 749 16800908

[B40] Staiger PK Serlachius A Macfarlane S Anderson S Chan T Young G Improving the coordination of care for low back pain patients by creating better links between acute and community services Aust Health Rev 2010 34 2 139 143 10.1071/AH08634 20497725

[B41] Grol R Successes and failures in the implementation of evidence-based guidelines for clinical practice Med Care 2001 39 8 Suppl 2 II46 II54 11583121 10.1097/00005650-200108002-00003

